# Current State of Cold Atmospheric Plasma and Cancer‐Immunity Cycle: Therapeutic Relevance and Overcoming Clinical Limitations Using Hydrogels

**DOI:** 10.1002/advs.202205803

**Published:** 2023-01-20

**Authors:** Milica Živanić, Albert Espona‐Noguera, Abraham Lin, Cristina Canal

**Affiliations:** ^1^ Biomaterials Biomechanics and Tissue Engineering Group Department of Materials Science and Engineering Escola d'Enginyeria Barcelona Est (EEBE) and Research Centre for Biomedical Engineering (CREB) Universitat Politècnica de Catalunya (UPC) c/Eduard Maristany 14 Barcelona 08019 Spain; ^2^ Biomaterials and Tissue Engineering Institut de Recerca Sant Joan de Déu Santa Rosa 39–57 Esplugues de Llobregat 08950 Spain; ^3^ Plasma Lab for Applications in Sustainability and Medicine‐Antwerp (PLASMANT) Department of Chemistry University of Antwerp Universiteitsplein 1 Wilrijk‐Antwerp 2610 Belgium; ^4^ Center for Oncological Research (CORE) Integrated Personalized & Precision Oncology Network (IPPON) University of Antwerp Universiteitsplein 1 Wilrijk‐Antwerp 2610 Belgium

**Keywords:** cancer, cold atmospheric plasma, drug delivery, hydrogels, immunogenic cell death, immunotherapy

## Abstract

Cold atmospheric plasma (CAP) is a partially ionized gas that gains attention as a well‐tolerated cancer treatment that can enhance anti‐tumor immune responses, which are important for durable therapeutic effects. This review offers a comprehensive and critical summary on the current understanding of mechanisms in which CAP can assist anti‐tumor immunity: induction of immunogenic cell death, oxidative post‐translational modifications of the tumor and its microenvironment, epigenetic regulation of aberrant gene expression, and enhancement of immune cell functions. This should provide a rationale for the effective and meaningful clinical implementation of CAP. As discussed here, despite its potential, CAP faces different clinical limitations associated with the current CAP treatment modalities: direct exposure of cancerous cells to plasma, and indirect treatment through injection of plasma‐treated liquids in the tumor. To this end, a novel modality is proposed: plasma‐treated hydrogels (PTHs) that can not only help overcome some of the clinical limitations but also offer a convenient platform for combining CAP with existing drugs to improve therapeutic responses and contribute to the clinical translation of CAP. Finally, by integrating expertise in biomaterials and plasma medicine, practical considerations and prospective for the development of PTHs are offered.

## Graphical abstract

Cold atmospheric plasma emerges as a cancer therapy that can activate and enhance anti‐tumor immune responses through different mechanisms. This ensures durable and systemic therapeutic effects. This review presents a comprehensive summary of immune‐response promoting mechanisms of plasma therapy, the limitations of current plasma modalities, and cutting‐edge developments in plasma‐treated hydrogels as a novel, minimally‐invasive modality to treat non‐surface tumors.

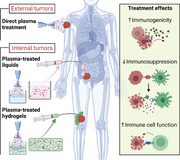

## 1 Background

Cold atmospheric plasma (CAP) is a partially ionized gas that represents a promising tool in biomedical research, with medically relevant applications ranging from wound healing and disinfection to cancer treatment.^[^ ^1^, ^2^ ^]^ Its clinical utility lies in the fact that CAP is an adjustable, exogenous source of reactive oxygen and nitrogen species (RONS).^[^ ^3^, ^4^ ^]^ RONS are known to act as pleiotropic signaling agents in the cells.^[^ ^5^, ^6^ ^]^ At physiological levels, RONS are involved in normal, healthy biological processes (e.g., cell proliferation and differentiation), but at more elevated levels, they can elicit detrimental and pathophysiological responses (e.g., cell death and malignancy).^[^ ^7^, ^8^, ^9^ ^]^ This implies that low concentrations of CAP‐generated RONS could be used to stimulate physiological signaling in cells for wound healing applications, whereas high concentrations could be used to induce cell death for cancer applications.

Cells endogenously generate RONS during cellular respiration in the mitochondria^[^ ^6^, ^7^, ^8^ ^]^ and during amino acid breakdown.^[^ ^9^ ^]^ Many features commonly found in cancer (e.g., increased metabolic activity, genome instability, and hypoxia) are associated with or rely on the increased generation and accumulation of endogenous RONS.^[^ ^10^, ^11^, ^12^ ^]^ This implies that compared to healthy cells, cancer cells have higher levels of endogenous RONS and may be more likely to surpass the cytotoxic RONS threshold and die when an exogenous source of RONS (such as CAP) is applied to them.^[^ ^13^, ^14^, ^15^ ^]^ In this sense, CAP is increasingly being studied as a cancer therapy with minimal cytotoxicity toward non‐malignant cells.^[^ ^16^ ^]^ Molecular mechanisms underlying the selectivity of CAP cancer therapy have been described in numerous articles, with the CAP‐generated RONS being identified as the main effectors.^[^ ^15^, ^16^, ^17^, ^18^, ^19^ ^]^ Nevertheless, not all cells respond equally to the CAP treatment.^[^ ^20^, ^21^, ^22^, ^23^ ^]^ To this end, there is an effort to understand the main underlying cellular response pathways as this could enable better prediction and improvement of CAP treatment outcome.^[^ ^24^, ^25^, ^26^ ^]^

For cancer treatment to be long‐lasting and efficient for different cancer types, it is often not sufficient that it solely provides cytotoxicity to cancer cells. Namely, cancer cells across and within the tumors are heterogeneous and might respond differently to the same treatment.^[^ ^27^ ^]^ The cells that survive the treatment or surgery often lead to tumor recurrence and metastasis. Thus, to ensure lasting tumor control and clearance on a systemic level (throughout the body), a cancer treatment should also promote anti‐tumor immunity. Anti‐tumor immunity is the entirety of immune cells and responses that ensure systemic and specific recognition and clearance of tumor cells. It can be achieved through induction of immunogenic cell death (ICD) in cancer, a type of regulated cell death that is recognized by the immune system.^[^ ^28^, ^29^, ^30^ ^]^ ICD helps initiate or enhance a series of events, known as the cancer‐immunity cycle,^[^ ^31^ ^]^ which results in anti‐tumor immunity. Namely, when cancer cells die by ICD, this promotes recruitment of antigen presenting cells (APCs) to the tumor site and provides adjuvancy. The cancer‐immunity cycle begins when these APCs capture tumor‐specific neoantigens released from dying cancer cells and transport them to draining lymph nodes, where they are used to prime and activate T cells. Primed T cells can then travel through the body to specifically find and kill tumor cells. However, different cancer properties such as a lack of neoantigens, overexpression of “don't eat me” signals, and an immunosuppressive tumor microenvironment (TME), can interfere with the cancer‐immunity cycle, resulting in low T cell activation and infiltration to tumor bed.^[^ ^32^, ^33^ ^]^ This calls for therapeutic approaches that can counteract these properties and thereby assist the cancer‐immunity cycle.

**Figure 1:**
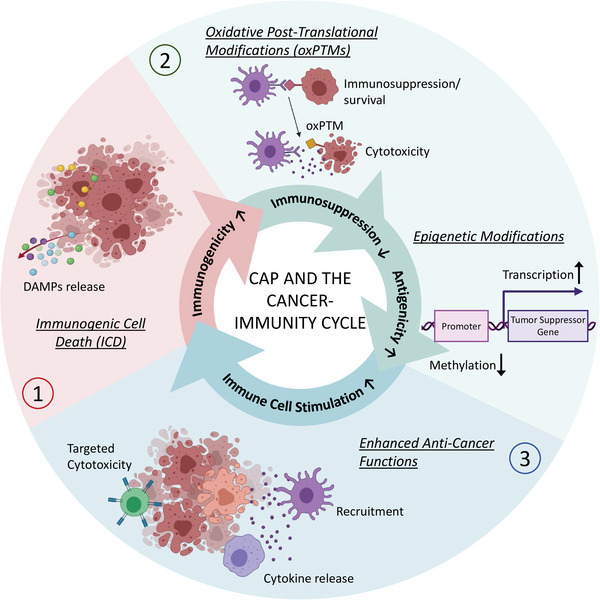
Different mechanisms in which CAP treatment could assist initiation of the cancer‐immunity cycle and subsequent immune responses. Figure created with Biorender.

In recent years, it has come to attention that CAP could both 1) help initiate the cancer‐immunity cycle (through induction of ICD) and 2) assist subsequent anti‐tumor immune responses (by promoting immune cells functions and counteracting immunosuppression through different mechanisms) (**Figure** **1**). Thus, there is promising evidence that CAP could be used as an immunomodulating agent or an adjuvant in cancer (immuno)therapies for enhanced therapeutic response.^[^ ^34^ ^]^ However, drawbacks associated with current CAP treatment modalities limit its clinical utility for different cancers. Currently, the two main CAP treatment modalities are 1) direct treatment, where the surface intended for the treatment (e.g., cancer cells or tissue) is directly exposed to the device that generates CAP, and 2) indirect treatment, where CAP is used to treat a liquid, which is then transferred to the treatment target. In the latter case, the CAP is only used to enrich a liquid with RONS (thus, obtaining a so‐called plasma‐treated liquid—PTL) and is never directly in contact with the treatment target. While both CAP modalities have been demonstrated to effectively elicit anti‐cancer and immunotherapeutic effects, they each have severe limitations for treatment of various cancer types. Namely, direct CAP treatment can be a highly precise therapy, but it requires unimpeded access to the tumor bed. Thus, this modality is limited to superficial tumors (e.g., melanoma, head and neck squamous cell carcinomas) or it otherwise necessitates more invasive procedures to access deeper tissue (e.g., laparoscopy or surgery). Alternatively, indirect treatment can be used to non‐invasively reach non‐superficial tumors via injection or perfusion of PTLs into the body. PTLs act as a carrier of CAP‐generated RONS but are difficult to control and are often quickly diluted by liquids within the body following their introduction.

To this end, plasma‐treated hydrogels (PTHs) could offer a novel and, in a way, hybrid form of direct and indirect CAP treatment. By integrating expertise in biomaterials and plasma for biomedicine, we recently proposed this modality as a promising alternative to work around the current limitations of CAP treatment methods.^[^ ^35^ ^]^ Use of PTHs could allow exploitation of the immunotherapeutic and anti‐cancer effects of CAP in a broader range of cancer types than what is currently possible. In addition, it could facilitate development of combinatorial therapies.

In this review, we discuss and provide a comprehensive summary of the current knowledge on the ability of CAP to drive the cancer‐immunity cycle and enhance anti‐tumor immunity through different mechanisms presented (Figure 1). We will discuss the limitations of current CAP treatment modalities, and how PTHs may offer a new, perspective method for precise and minimally‐invasive treatment of non‐superficial tumors within the body. In the context of hydrogel‐based drug delivery and tissue‐engineering, the exciting and new possibilities for advanced CAP‐based treatment strategies using PTHs will be put into perspective. The insights presented in this review could be used for the development of more rational and effective combination strategies with current immunotherapies and the advancement of clinical translation of CAP technology.

## 2 CAP Treatment Promotes Anti‐Tumor Immunity

Therapies that promote anti‐tumor immune responses for cancer clearance and control have revolutionized the field of oncology, enabling robust and durable effects across different cancer types.^[^ ^36^ ^]^ There is increasing evidence that CAP could enhance the anti‐tumor immune responses through several different mechanisms, making it a promising (adjuvant) cancer therapy. These can be summarized in four different categories, as follows:

1) Induction of Immunogenic Cell Death (ICD) in Cancer:
  During ICD, dying cells display damage‐associated molecular patterns (DAMPs), which promote inflammatory responses and act as adjuvants to facilitate the recruitment and activation of APCs. In this way, CAP increases the visibility of cancer cells to the immune cells and helps initiation of the cancer‐immunity cycle.
2) Introduction of Oxidative Post‐Translational Modifications (oxPTMs) to Proteins and Peptides:
  oxPTMs increase the repertoire of tumor‐specific antigens (neoantigens), which enhances T cell priming and activation. In addition, through oxPTMs, physical, chemical, and functional properties of proteins including protein–protein interactions can be altered. Thus, CAP can alter the interaction among cancer cells, the TME, and immune cells, to lessen tumor invasiveness and immunosuppression.
3) Epigenetic Modification of Aberrant Gene Expression:
  Through epigenetic modifications, tumor cells can regulate the expression of genes to aberrate the normal cell cycle and escape immune responses. For example, cancers can downregulate tumor‐suppressors and ICD‐markers and upregulate oncogenes. As epigenetic changes are reversible, epigenetic drugs can be used to restore the abnormal gene expression in cancer to increase their sensitivity to the treatment and/or immune responses. While the investigation into CAP‐induced epigenetic modifications is still limited, there is evidence that suggests CAP could promote anti‐cancer effects via this pathway.
4) Enhancement of the Anti‐Tumor Functions of Immune Cells:
  Immune cells can respond to CAP with altered chemokine/cytokine secretion and expression of cellular markers or phenotypic changes in a cell‐type specific manner (e.g., macrophages, dendritic cells, T cells). This can promote their pro‐inflammatory and anti‐cancer functions for enhanced tumor killing.

Through these mechanisms, CAP can achieve the following:

- Enhance immunogenicity (visibility of cancer cells to immune cells)
- Enhance antigenicity (the degree to which a cancer cell differs from a non‐malignant cell and can as such be recognized by immune cells)
- Enhance the anti‐tumor capacity of immune cells (the ability of the immune system to fight cancer)
- Reduce immunosuppression (the ability of a tumor to evade immune cells infiltration and responses)
- Reduce tumorigenicity (aberrant properties of cancer cells that promote their invasiveness and resistance to treatment and immune responses)

### 2.1 CAP Treatment Induces Immunogenic Cell Death in Cancer

When cancer cells undergo ICD, they display a variety of endogenous signals, known as damage‐associated molecular patterns (DAMPs), which act as adjuvants to facilitate recruitment and maturation of local immune cells.^[^ ^29^, ^30^, ^37^ ^]^ This helps initiate the patient's cancer‐immunity cycle for the development of a robust immune response against the cancer.^[^ ^31^ ^]^ DAMPs are actively or passively secreted from the cell (e.g., adenosine‐triphosphate [ATP], high mobility group box 1 protein [HMGB1]) or are translocated from the inside of the cell to the cell membrane (e.g., calreticulin [CRT], heat shock proteins [HSPs]).^[^ ^29^, ^37^ ^]^ Thus, the most simple and common way to evaluate ICD‐inducing potential of a therapy is to detect and quantify the presence of different DAMPs in vitro via flow cytometry, enzyme‐linked immunosorbent assay (ELISA), and others.^[^ ^38^ ^]^ However, it is important to note that in vivo ICD‐associated DAMPs could be inhibited through the presence of inhibitory DAMPs or other neutralization pathways.^[^ ^39^, ^40^, ^41^ ^]^ The expression of DAMPs can also be regulated epigenetically^[^ ^42^ ^]^ or can correlate to the expression status of further molecules.^[^ ^43^ ^]^ Thus, differential DAMPs expression can be found for different tumors. Therefore, detecting DAMPs in vivo, in the context of different cancer types and TMEs is of high importance.

**Figure 2:**
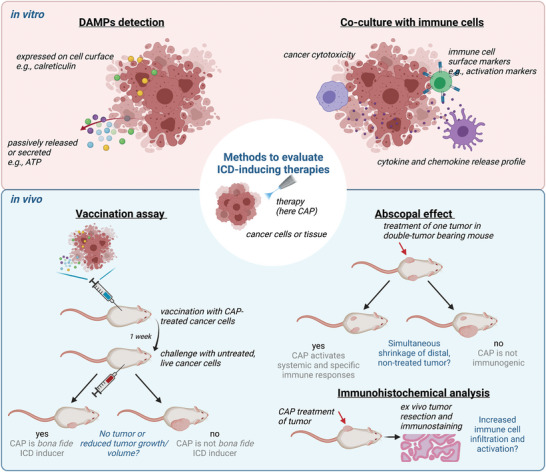
Different in vitro and in vivo methods to characterize the ability of the treatment (here CAP) to induce immunogenic cell death (ICD) in cancer and drive systemic and specific anti‐tumor immune responses (anti‐tumor immunity). Untreated cancer cells or mice are used as a negative control, and a known ICD‐inducing drug (e.g., Mitoxantrone) can be used as a positive control. Figure created with Biorender.

Overall, DAMPs are considered surrogate ICD markers, whereas the vaccination assay represents the gold‐standard method for evaluation of ICD‐inducing therapies (**Figure** **2**). Thus, following the successful completion of the vaccination assay, a therapy can be considered a bona fide inducer of ICD.^[^ ^28^ ^]^ In this assay, cancer cells are treated with a potential ICD‐inducer and then injected into a healthy, syngeneic mouse, in essence like a whole‐cell vaccine.^[^ ^44^ ^]^ A week later, the mouse is challenged with the same, but live, untreated cancer cells in an area different to the vaccination site. If the vaccinated mice are more protected against tumor development compared to those in the control group, the therapy is considered to act as an ICD‐inducer and to be able to drive specific and systemic anti‐tumor immunity. There are also further therapeutic effects that can be seen as indicators of ICD‐inducing ability, such as an increased activation and infiltration of immune cells in the treated tumor, immunomodulation, and abscopal effects (Figure 2). The abscopal effect describes shrinkage of an untreated tumor together with the shrinkage of the treated tumor. This is likely to be mediated through induction of ICD in the treated tumor, which then promotes antigen presentation and activation of the adaptive immune system. The adaptive immunity acts on a systemic level; and is thus, able to recognize; and thus, clear distal, non‐treated tumors.

In the recent years, CAP has been recognized as an ICD‐inducer^[^ ^45^ ^]^ through detection of DAMPs, the vaccination assay, observation of abscopal effects, and stimulative effects on the functions of immune cells (**Table** **1**). In biomedical research, CAP is most commonly generated using dielectric barrier discharges (DBD) and atmospheric pressure plasma jets (APPJ), including the medically certified and commercially available kINPen APPJ device.^[^ ^46^, ^47^ ^]^ Most of the research on CAP and ICD has been done for the direct CAP treatment and there are notably less studies that evaluated the ability of indirect CAP treatment to drive ICD and anti‐tumor immunity. PTLs employed in these studies were plasma‐treated PBS (pPBS)^[^ ^48^, ^49^ ^]^ or cell culture medium (PTM).^[^ ^50^, ^51^ ^]^ For indirect CAP to become a viable clinical treatment, the liquid used for CAP enrichment must be clinically approved and carefully evaluated. To this end, Freund, et al. had analyzed six different clinically approved liquids treated with CAP. The authors reported, that among different PTLs, sodium chloride showed the highest consistency in terms of both RONS stability and anti‐tumor effects in the CT26 colorectal cancer cell line.^[^ ^52^ ^]^ However, the ICD‐inducing ability of different clinically approved PTLs is yet to be characterized.

**Table 1:** CAP was demonstrated to induce immunogenic cell death (ICD) in various cancer types using different evaluation methods (DAMPs detection, vaccination assay, abscopal effect, and immunomodulatory effects)

Cancer type	Cell line	Indicators of ICD	CAP treatment modality, CAP source (and feed gas)	Ref.
Breast	MCF‐7, MDA‐MB (human, 2D and 3D spheroid tumor models) 4T1 (murine, in vivo)	**In vitro**: CRT ↑, HSP70 ↑, HSP90 ↑, MHC‐I ↑, pro‐inflammatory cytokines ↑, but PD‐L1 ↑ (*ICD‐markers were elevated at 24 h, but even more so at 48 h post‐treatment*) **In vivo**: T‐cells and DCs infiltration ↑, abscopal effect	Direct, APPJ (He)	[53]
Leukemic	Jurkat (human, T‐lymphocytes) (*Study compared biological responses with THP‐1* *human* *monocytes*)	**In vitro**: CRT ↑, HSP70 ↑, HSP90 ↑ Monocyte migration ↑ and phagocytosis by macrophages ↑ (*In contrast to Jurkat, for THP‐1 CAP had little effect on ICD markers and viability, but still CAP‐treated THP‐1 cells promoted immune cells functions*)	Direct, DBD (*ICD indicators were reduced using intracellular ROS attenuator NAC; cells altered RONS composition in medium in cell‐specific manner*; *THP‐1 cells immediately depleted* *H*_*2*_ *O*_*2*_)	[21]
Melanoma	B16F10 (murine) (*The first study to trace all the different stages of the cancer‐immunity cycle by analyzing both tumor bed and lymph organs*)	**In vivo**: CRT ↑, PD‐L1 ↓, but no change for CD47 DCs infiltration ↑, antigen presentation ↑, T cell activation, and cytotoxic activity ↑ (*Note that many of these effects were transient and were the most pronounced at day 10*, *which is 3 days after the final CAP treatment*)	Direct, DBD	[54]
	B16F10 (murine)	**In vitro**: CRT ↑, HSP90 ↑, but CD47 ↑ and no change for MHC‐I **Vaccination assay**: No animals developed tumor at vaccination site 50% (3/6) of C57BL/6J mice did not develop tumor (compared to 5/6 mice vaccinated with MTX‐vaccine) **In vivo**: Immune cells infiltration ↑, T‐cells activation ↑	Direct, kINPen (Ar, Ar/O_2_, He, He/O_2_) (*Characterized cytotoxicity and immunomodulatory effects of CAP using different carrier gases (which affect RONS composition and concentration); in this study, Ar* (*high H*_2_ *O*_2_) *and He/O*_*2*_ *(high HOCl)* *were optimal*)	[55]
	A375 (human) B16F10 (murine)	**In vitro**: CRT ↑ **Vaccination assay**: No animals in different treatment groups developed tumor at vaccination site 62.5% (5/8) of C57BL/6J mice did not develop tumor (compared to 5/8 mice vaccinated with MTX‐vaccine)	Direct, DBD (*Discussed the importance of short‐lived RONS for ICD induction and delineated the effect of long‐lived RONS and pulsed electric field*)	[56]
	B16F10 (murine)	**In vitro**: ATP ↑, CXCL‐1 ↑, pro‐inflammatory cytokines/chemokines release ↑ Monocyte‐to‐macrophage differentiation ↑, altered secretory profile of splenocytes (inconclusive) in co‐culture	Direct, kINPen (Ar)	[57]
	B16F10 (murine)	**In vitro**: CRT ↑, MHC‐I ↑, MC1R ↑, VEGF ↓	Direct, kINPen (Ar)	[58]
	B16F10 (murine)	**In vivo**: Abscopal effect	Direct, streamer discharge	[59]
	HBL, Hmel1 (human)	**In vitro**: CRT ↑, ATP ↑	Indirect (PTM), DBD (*Observed H*_*2*_ *O*_*2*_ *as main effector; observed different cell death mechanisms in melanoma (autophagy) and pancreas (apoptosis) cancer cells*)	[50]
Pancreas	Cancer cells: MIA‐Paca2, PANC‐1, BxPC3, Capan‐2 Stellate cells: hPSC128, hPSC21, RLT‐PSC (all human)	**In vitro**: CRT ↑, ATP ↑, HMGB1 ↑, CD47 ↓ Phagocytosis by DCs ↑, maturation of DCs ↑, pro‐inflammatory cytokines ↑ in co‐culture (*These effects were less pronounced in stellate cells, but still present, which is an important observation as these cells are known to be immunosuppressive*)	Indirect (pPBS), kINPen (Ar)	[49]
	PDA6606 (murine)	**In vivo**: CRT ↑, immune cell infiltration ↑	Indirect (PTM), kINPen (Ar)	[51]
Colorectal	CT26 (murine)	**In vivo**: Abscopal effect (*However, abscopal effect was also observed upon treatment of normal tissue*)	Direct, streamer discharge	[60]
	CT26 (murine) (*Study also observed upregulation of DAMPs in MC38 and PDA6606 cells, and no upregulation in non‐malignant HaCat cell line for same CAP treatment*)	**In vitro**: CRT ↑, HSP70 ↑, HMGB1 ↑, nuclear/cytosolic HMGB1 ratio ↑, pro‐inflammatory cytokine profile ↑, but ATP ↓ **In vivo**: Macrophage count ↑, T‐cell activation ↑	Indirect (pPBS), kINPen (Ar) (*Showed that presence of H*_*2*_ *O*_*2*_ *degrading enzyme catalase abolishes increase in ICD‐markers on cell surface*)	[48]
	CT26 (murine)	**In vitro**: CRT ↑, ATP ↑ **Vaccination assay**: 33.3% (3/10) Balb/c mice did not develop tumor and 90% of mice had reduced relative mean tumor volume **In vivo**: CRT ↑, HMGB1 ↑, immune cell infiltration ↑, specific T‐cell response ↑	Direct, DBD (*Showed in subcutaneous tumor that treatment of multiple spots over 5 days could be a good strategy for induction of ICD in vivo*)	[61]
	CT26 (murine)	**In vitro**: CRT ↑ (both on dead and still viable cells)	Direct, kINPen (Ar)	[62]
	CT26 (murine) (*With cellular or acellular ECM‐like barrier*)	**In vitro**: CRT ↑, ATP ↑	Direct, DBD (*Showed that (direct) cell–cell signaling assists CAP effect propagation and penetration depth*)	[63]
Glioblastoma	LN‐229, U‐87, T98G (human)	**In vitro**: CRT ↑, ATP ↑, HMGB1 ↑ DCs maturation (CD86 marker) ↑, but DC‐mediated phagocytosis ↓ (due to auranofin)	Indirect (pPBS), kINPen (Ar); sequential combination with auranofin (*Cell death via both apoptotic and ferroptotic mechanisms; cell‐type specific treatment sensitivity correlated with baseline protein levels of antioxidant system*)	[64]
Lung	A549 (human)	**In vitro**: CRT ↑, ATP ↑ Macrophages killing capacity ↑	Direct, DBD (*ICD indicators were reduced using intracellular ROS attenuators NAC and DPI and the physical components of CAP (e.g., pulsed‐electric fields, streamer electric field, and UV radiation) did not stimulate ICD* )	[65]
Naso‐pharyngeal	CNE‐1 (human, radiation‐resistant)	**In vitro**: ATF4 and STC2 (located upstream of CRT) ↑, ATP ↑ Macrophages killing capacity ↑	Direct, DBD	[66]
Rhabdomyosarcoma	MX‐7 (murine)	**In vitro**: CRT ↑, HSP70 ↑, HMGB1↑ **In vivo**: Blood serum level of HMGB1↑ (increased only in C3H/He tumor‐bearing mice and not in healthy mice) (*In addition, cytokines G‐CSF ↑, IL−4 ↓ in healthy mice*; *CAP can still be considered immuno‐safe treatment; although, further investigation would be useful*)	Direct, APPJ (He)	[67]

Abbreviations: CRT, calreticulin; HSP, heat shock protein; MHC‐I, major histocompatibility complex class I; PD‐L1, programmed death‐ligand 1; DCs, dendritic cells; APPJ, atmospheric pressure plasma jet; DBD, dielectric barrier discharge; kINPen, commercially available APPJ; NAC, N‐acetyl cysteine; MTX‐vaccine, vaccine obtained by treating cancer cells with known immunogenic drug mitoxantrone; CXCL1, C‐X‐C motif chemokine ligand 1; MC1R, melanocortin 1 receptor; VEGF, vascular endothelial growth factor; ATP, adenosine triphosphate; HMGB1, high mobility group box 1 protein; DPI, diphenyleneiodonium; ATF4, activating transcription factor 4; STC2, stanniocalcin 2; PTM, plasma‐treated medium; pPBS, plasma‐treated PBS.

As can be seen from Table 1, the vaccination assay is only beginning to be included for ICD evaluation, and usually the studies employ direct CAP treatment to obtain the whole‐cell vaccine for the assay.^[^ ^55^, ^56^, ^61^ ^]^ For successful completion and interpretation of the vaccination assay, it is critical that injected cancer cells are fully inactivated and unable to develop tumors at the vaccination site. Namely, such tumors would not only contribute to the total tumor burden but also cofound immune responses associated with the vaccination. Lin et al. reported an optimized vaccination protocol for inactivation of melanoma using the DBD plasma, which could be translated to both direct and indirect CAP treatment.^[^ ^56^ ^]^ Namely, they included an additional step of incubating the whole‐cell vaccine in PBS at 37 °C, to significantly limit the number of animals that developed tumors at the vaccination site. Such vaccine showed an efficacy of 62.5% (5/8 mice), which was comparable to the positive vaccine control generated using a well‐known ICD‐inducer, Mitoxantrone.^[^ ^56^ ^]^ Similar results, also for melanoma, were recently achieved by Bekeschus et al. for the vaccine generated with the kINPen (using Ar gas), with no animals developing tumors at the vaccination site and a vaccine efficacy of 50%.^[^ ^55^ ^]^ Reporting such detailed, optimized vaccination protocols for different CAP sources represents an important step toward research standardization and identification of commonality between devices and modalities.

Importantly, so far, abscopal effect was observed upon direct CAP treatment of different murine tumors including breast,^[^ ^53^ ^]^ melanoma,^[^ ^59^ ^]^ and colorectal.^[^ ^60^ ^]^ Even the treatment of normal tissue with CAP led to shrinkage of distant colorectal carcinoma in mice.^[^ ^60^ ^]^ These results could be of interest from the perspective of ease of clinical application of CAP treatment, but they require critical characterization to determine and compare the mechanisms of healthy‐ and cancer tissue‐mediated abscopal effects in the context of adaptive immune system activation and clinical safety.

### 2.2 CAP Treatment Introduces Oxidative Post‐Translational Modifications to the Tumor and Microenvironment

RONS from CAP can oxidize biomolecules and introduce diverse oxidative post‐translational modifications (oxPTMs) to a protein.^[^ ^68^, ^69^ ^]^ oxPTMs can alter physical, chemical, and functional properties of the protein, including solubility, localization, folding, and interaction with other biomolecules. This can have implications for different cellular pathways. For example, CAP‐induced oxidation of ASK1:TRX1 could disrupt the interaction of these proteins to promote pro‐apoptotic signaling pathways.^[^ ^70^ ^]^ Here, we summarize the different effects of CAP‐induced oxPTM of proteins and their implications for cancer therapy (**Table** **2**).

**Table 2:** CAP induces oxidative post‐translational modifications (oxPTMs) of biomolecules with therapeutic relevance in cancer treatment

Effect of oxPTM	Biomolecule	Significance in cancer treatment	Ref.
Antigenicity ↑	Ovalbumin (Ova) (*Egg white protein, used as model protein to study activation of anti‐Ova T cells*)	Anti‐tumor vaccine with augmented repertoire of neoantigens for enhanced T cell activation (*Here, the best results were achieved using helium/oxygen plasma*, *a carrier gas that favored generation of HOCl*)	[71, 72]
Activity ↑	Lysozyme (*Secretory product of macrophages with anti‐tumor effect*)	Enhanced cancer cytotoxicity	[73, 74]
Binding affinity ↓	Hyaluronan (HA), CD44 (*Their interaction promotes cell proliferation, invasion, and metastasis*)	Inhibition of tumor progression and metastasis	[75]
	CD47 (*Binds to SIRP* α *immune cell receptor to provide* “*do not eat me*” *immunosuppressive signal*)	Counteracting immunosuppression in tumor	[76]
	ASK1:TRX1 (*TRX1 serves as a regulatory redox switch, which when oxidized dissociates from ASK1 to allow activation of p38 and pro‐apoptotic pathways*)	Activation of pro‐apoptotic pathways (activation of stress signaling)	[70]
Structural integrity and function ↓	Haemoglobin, myoglobin (*Oxygen‐carriers*)	Considerations for safe and effective clinical translation (e.g., plasma carrier gas and composition, plasma application site) (*RONS can modify blood proteins and blood can have antioxidant and scavenging effect on RONS; differences between air, nitrogen (strongest effect) or argon plasma; further factors beside* *H*_*2*_ *O*_*2*_ *concentration play role*)	[77, 78]
	SIRT6, COX2 (*Enzymes that are common targets in skin cancer treatment*)	Combination therapy in skin cancer	[79]

Abbreviations: CD, cluster of differentiation; SIRP*α*, signal regulatory protein *α*; ASK1, apoptosis signal‐regulating kinase 1 also known as mitogen‐activated protein kinase 5 (MAP3K5); TRX1, thioredoxin 1; SIRT6, sirtuin 6; COX2, cyclooxygenase‐2.

Through oxPTMs, CAP can augment a repertoire of tumor‐specific antigens and confer higher antigenicity to the tumor ligands and receptors. As tumor antigens are required for effective T cell priming by APCs, CAP‐mediated oxPTMs could enhance anti‐tumor immunity.^[^ ^71^, ^72^ ^]^ To this end, Clemen et al., reported that mice vaccinated with CAP‐treated ovalbumin protein (oxOva) showed higher immune responses and protection against Ova‐expressing melanoma cells compared to mice that were vaccinated with untreated ovalbumin (Ova).^[^ ^71^ ^]^ In their study, they compared the efficacy of two different carrier gases for the generation of CAP and reported a helium and oxygen mixture (He/O_2_) to be particularly effective for oxidizing ovalbumin proteins and activating T cells, compared to argon (Ar).^[^ ^71^ ^]^ This could be due to the difference, where the He/O_2_ carrier gas favored generation of hypochlorous acid (HOCl), whereas the argon carrier gas favored H_2_O_2_ generation.^[^ ^55^, ^71^ ^]^ Namely, HOCl is known to act as a natural adjuvant of adaptive immunity and is used by neutrophils to oxidize proteins.^[^ ^80^, ^81^ ^]^ Furthermore, HOCl has been successfully used to generate dendritic cells (DC)‐based cancer vaccines with oxidized whole‐tumor lysate.^[^ ^82^, ^83^, ^84^ ^]^

oxPTM of cancer cell receptors and proteins in their extracellular environment can also diminish their tumorigenic and immunosuppressive functions. A recent study employing cells from different cancer tissues (glioblastoma, melanoma, and colorectal cancer) provided experimental and computational evidence that CAP can oxidize hyaluronan (HA) and CD44, thereby reducing the proliferative capacity of cancer cells.^[^ ^75^ ^]^ Namely, HA is a polysaccharide abundantly present in the extracellular matrix and CD44 is an important cell adhesion receptor, often overexpressed in cancer, that facilitates the interaction with different components of the TME. These molecules promote cancer proliferation, migration, adhesion, and inflammatory processes; and thus, can be therapeutically targeted in cancer.^[^ ^85^ ^]^ Exposure to CAP was also shown to downregulate CD44 on a transcriptional level. This was observed in breast cancer cell lines with different metastatic potentials.^[^ ^86^ ^]^ The study showed that CAP treatment also modulates mRNA levels of a few other molecules involved in cancer tumorigenesis and metastasis (e.g., metalloproteinases, which can promote cancer invasion and metastasis). Unlike CD44, mRNA levels of other molecules were modulated in a cell‐type dependent manner as they were upregulated in estrogen‐positive cells (low‐metastatic potential) and downregulated in estrogen‐negative cells (high‐metastatic potential). Altogether, the findings of this study further imply that the regulation of different microenvironmental effectors is one of the important mechanisms behind CAP anti‐tumor effects.^[^ ^86^ ^]^

CAP was also investigated for its ability to oxidize CD47, another cellular receptor commonly overexpressed in cancer. CD47 is an important immune checkpoint which inhibits the function of innate immune cells, particularly DCs. Direct CAP treatment of 3D spheroids and in ovo tumor models of different human cancers led to decreased detection of CD47 via flow cytometry, while in the mouse model, this decrease was not significant.^[^ ^76^ ^]^ As the decrease happened immediately after exposure to CAP, a plausible possibility is that CAP oxidized CD47 and induced conformational changes to it, which resulted in decreased binding of CD47 with the antibody used for staining in flow cytometry. The authors supported the hypothesis through in silico simulations. Using molecular dynamic simulations and docking studies, the authors investigated the binding of (oxidized) CD47 with its immune receptor, the signal regulatory protein *α* (SIRP*α*). The simulations revealed that oxidation of CD47 decreased its binding affinity, most likely through oxidation of specific salt‐bridges that led to conformational changes in the receptor protein.^[^ ^76^ ^]^ Importantly, this study employed the same direct CAP treatment regimen that previously induced ICD in the same melanoma cells.^[^ ^56^, ^76^ ^]^ This suggests that CAP therapy can simultaneously increase immunogenicity and decrease immunosuppression to promote anti‐tumor immunity. Curiously, in several other studies, exposure to CAP had no effect or even upregulated CD47 expression in different cancer types (leukemia^[^ ^21^ ^]^ and murine melanoma,^[^ ^55^ ^]^ respectively); though, these studies still reported that the CAP treatment had immunogenic and immune‐promoting effects. As CD47 is just one of many microenvironmental factors in tumor immunosuppression, the final therapeutic effects could depend on a balance of multiple tumor‐expressing signals. Furthermore, the time‐point at which the protein is quantified is critically important, as oxPTMs could be transient. For example, in the 3D glioblastoma and head and neck squamous carcinoma spheroid models, detection of CD47 was reduced immediately after CAP exposure but was restored to baseline levels at 24 h after the exposure. On the other hand, in the in ovo melanoma model, this reduction persisted for 24 h after the exposure.^[^ ^76^ ^]^

Interestingly, indirect CAP treatment also led to a decreased detection of CD47 on cancer cell surface. This was observed 48 h after the treatment of several different pancreatic cancer cell (PCC) and immunosuppressive pancreatic stellate cell (PSC) lines and was accompanied with increased phagocytosis and maturation of DCs.^[^ ^49^ ^]^ At the same time, such treatment was able to induce ICD in PCC.^[^ ^49^ ^]^ Nevertheless, the mechanisms by which direct and indirect CAP treatment lessen the immunosuppression in cancer might be different. In a recent effort to identify the main RONS effectors in CAP‐mediated oxidation of biomolecules, Wenske and his colleagues reported short‐lived species and secondary species (particularly HOCl and ONOO^−^) to be of the greatest importance regarding oxidation of peptides.^[^ ^68^ ^]^ To this end, it is of interest to investigate for each of the two CAP treatment modalities, the main pathways and RONS molecules involved in modulation of the tumor microenvironmental effectors.

### 2.3 CAP Treatment Could Epigenetically Regulate Aberrant Gene Expression in Tumors

Epigenetic modifications are reversible, heritable changes of the genome (in the promoter regions of DNA or on histone proteins) that do not influence nucleotide sequence but can modify the activity of genes.^[^ ^87^ ^]^ Epigenetic changes are relevant for tumorigenesis, immunosuppression, and cancer heterogeneity (including cancer stem cells population) as they can cause aberrant expression of oncogenes, tumor suppressor genes, immune checkpoints, chemokines, and so on.^[^ ^88^ ^]^ Their reversibility makes epigenetic changes a promising treatment target, and drugs targeting epigenetic markers (epi‐drugs) are increasingly receiving attention for cancer treatment. Usually, such epi‐drugs aim to recover gene expression (e.g., of tumor suppressor genes such as cell cycle checkpoints) that was silenced through DNA methylation or histone deacetylation and closing of chromatin.^[^ ^88^, ^89^ ^]^ There is also an emerging class of epi‐drugs that targets histone methylation, which is a more complex epigenetic modification that can have both activating and repressing effects on gene expression.^[^ ^90^ ^]^ There are already several (marketed) epi‐drugs that showed promising anti‐tumor effects alone or in combination with other cancer therapies, where they could reduce cancer stemness and drug‐resistance.^[^ ^88^, ^89^ ^]^

As epigenetic changes are known to be closely related to intracellular redox biology,^[^ ^91^, ^92^, ^93^, ^94^ ^]^ it can be expected that exposure of cells to CAP‐generated RONS can lead to some epigenetic modifications. Importantly, genome‐wide studies performed so far suggest that CAP‐induced epigenetic modifications are not on the global level and there is no evidence for safety concerns.^[^ ^95^, ^96^ ^]^ The role of CAP as an epigenetic modulator has not been often studied, but there are a few examples that indicate that CAP could modulate DNA methylation and histone methylation status to promote overall anti‐tumor effects (**Table** **3**). For example, CAP treatment of breast cancer, was shown to promote histone demethylation to downregulate several tumor‐associated genes, such as PRSP1, which is known to be overexpressed in cisplatin drug‐resistant breast cancer cell lines.^[^ ^95^ ^]^ Still, little is known about the mechanism, biological significance, or relevance of CAP‐induced epigenetic modifications in cancer. Recently, epigenetic effects of CAP treatment were also studied for healthy stem cell populations (adipose tissue derived stem cells) in the context of regenerative medicine, and CAP was reported to activate expression of different cytokines and growth factors.^[^ ^97^ ^]^ In this regard, it would be interesting to characterize the (epigenetic) effect of CAP also on cancer stem‐like cells (CSLC) populations. There is first evidence that suggests CAP treatment could favor the survival and expression of stemness genes in the metastatic CSLC in osteosarcoma;^[^ ^23^ ^]^ and thus, might need to be combined with further (epi‐)drugs for the complete therapeutic effect.

**Table 3:** CAP epigenetically regulates gene expression with therapeutic relevance in cancer treatment

Epigenetic change	Biomolecule	Significance in cancer treatment	Ref.
DNA methylation (Affects binding of transcription factors) Methylation ↑ − gene ↓ Methylation ↓ − gene ↑	Alu and Line‐1 transposable DNA elements, genome‐wide methylation analysis (*Studied in breast cancer cells: MCF‐7 and MDA‐MB‐231*)	Regulation of aberrant gene expression in tumor (e.g., upregulation of tumor‐suppressor genes and downregulation of oncogenes) (*Here, for example, an apoptosis regulating protein BCL2 and tumorigenic protein BDNF were hypermethylated and downregulated; effects were cell‐type and methylation‐site specific*)	[96]
	miR‐19 Methylation ↑ (*Oncogenic micro‐RNA that mediates cell proliferation*)	Suppression of cancer cell proliferation In addition, activation of tumor suppressors that are targets of miR‐19	[98]
	DUOX2 Methylation ↓ (*Elevates intracellular ROS levels together with NOX1 and NOX5 enzymes* *that were also upregulated upon CAP treatment*)	Enhanced levels of intracellular ROS (*Increased oxidative stress*) (*TET1 DNA demethylase as the main effect mediator*)	[99]
Histone methylation (Affects DNA packaging; and thus, accessibility of genes for transcription) For example, H3K4me3 ↓ − gene ↓	Different genes including HSCB and PRPS1 H3K4me3 ↓ (*Oncogenes important for colony formation in cancer*)	Inactivation of oncogenes (*JARID1A histone demethylase as the main effect mediator*; *Histone demethylases are emerging as targets in cancer therapy* [100])	[95]

Abbreviations: Line1, long interspersed nuclear elements; BCL2, B‐cell lymphoma 2; BDNF, brain‐derived neurotrophic factor; DUOX2, dual oxidase 2; NOX, nicotinamide adenine dinucleotide phosphate (NAPDH) oxidase; TET1, ten‐eleven translocation methylcytosine dioxygenase 1; HSCB, also called hHSC20, human heat shock cognate protein 20; PRPS1, phosphoribosyl–pyrophosphate synthetase 1; H3K4me3, a type of epigenetic modification – tri‐methylation at the fourth lysine residue of the histone H3 protein; JARID1A, Jumonji/ARID domain‐containing protein 1A.

There are several further potential research topics relating to CAP as epigenetic regulator. For example, regulation of many ICD hallmarks (DAMPs, pro‐inflammatory cytokines) has recently been brought into relation with epigenetic changes. Moreover, several epi‐drugs were reported to induce the expression of different DAMPs.^[^ ^42^ ^]^ To this end, it would be interesting to investigate the ability of CAP to promote ICD also through epigenetic mechanisms. Here, it is important to mention that epigenetic regulation of ICD hallmarks in different cancers can be both beneficial and disadvantageous; and thus; should be appropriately targeted in a therapeutic context. For example, compared to their healthy counterparts, in some cancer cells, a microRNA that inhibits the release of HMGB1 is downregulated through hypermethylation, which promotes the release of this ICD marker. Thus, it wouldn't be beneficial if a therapy recovered the expression of this microRNA through its demethylation. On the other hand, promoter regions of different pro‐inflammatory interleukins (e.g., IL‐1*β*) can also be hypermethylated and downregulated in cancer. Thus, such cancers could be suitable for demethylating epi‐drugs.^[^ ^42^ ^]^ Epi‐drugs are increasingly being recognized for their potential in counteracting immune suppression in cancer and TME and promoting immune responses. For example, there is evidence that they could be used to skew macrophage polarization toward more pro‐inflammatory phenotype.^[^ ^101^ ^]^ Taken together, it would be interesting to study potential synergistic effect of CAP and epi‐drugs. Last of all, it has been suggested that epigenetic signatures of cancer cells and associated immune cells could even be used as therapeutic predictors or biomarkers as they greatly contribute to the treatment outcome.^[^ ^102^ ^]^ Therefore, studying the effect of CAP on different candidate epigenetic biomarkers might help further understand the potential adjuvant role of CAP in immunotherapies.

### 2.4 CAP Treatment Enhances Immune Cell Functions

Through the aforementioned mechanisms (ICD‐induction, oxidative and epigenetic modifications of tumor), CAP can increase the tumor's antigenicity and immunogenicity and decrease its tumorigenic and immunosuppressive properties. These effects facilitate recruitment, activation, and infiltration of immune cells to promote cancer clearance and control. Beside indirect immune system stimulation through CAP treatment of cancer, CAP can also be used to treat immune cells and act as a direct immunomodulator. When immune cells are exposed to CAP, they can respond with phenotypic changes (e.g., differentiation and polarization of immune cells, receptor expression), metabolic changes, and altered chemokines/cytokines secretion profiles. This can consequently further augment the overall anti‐tumor and pro‐inflammatory immune responses. The immunomodulating effects of CAP on treated immune cells can be explained in part by the fact that immunometabolism is largely regulated by redox chemistry (RONS).^[^ ^103^ ^]^ Importantly, although the effects of CAP are overall immuno‐stimulatory, CAP treatment does not seem to lead to side effects or significant systemic changes in concentrations of cytokines and cells in blood.^[^ ^104^ ^]^

In summary, the stimulatory effects of CAP on anti‐tumor functions of immune cells can be observed when both cancer cells and immune cells are treated. These effects are usually studied by co‐culturing immune cells with cancer cells (in vitro), or by immunohistochemical analysis of CAP‐treated tumors (in vivo). However, in vivo, in the context of the TME that contains both cancer and immune cells, the evaluation of CAP treatment effect is much more complex, as delineation of immune cell treatment versus tumor cell treatment during exposure of the tumor to CAP is not possible. Even the use of immunodeficient mice will not be able to distinguish the direct effect of CAP on immune cells compared to the effect mediated by CAP‐treated tumor cells. In this context, it might be interesting to inject (ex vivo) CAP‐treated immune cells into syngeneic tumor‐bearing mice to evaluate their response, similar to how current DC‐cancer vaccines are being prepared. With the growing number of studies investigating CAP effect on immune cells, it will be important to have a more standardized research setting in the future that would facilitate clinical translation,^[^ ^105^ ^]^ similar to what has been reported for direct and indirect CAP treatment of standard cancer cell cultures and their molecular analysis.^[^ ^106^ ^]^

Naturally, different types of immune cells show different sensitivities and responses to direct CAP exposure, and this further depends on other factors, such as the cell donor, whether there has been prior stimulation of the cell with a mitogen, and other environmental factors.^[^ ^57^, ^107^, ^108^, ^109^ ^]^ Our knowledge on sensitivity and responses of immune cells to direct and, even more so, indirect CAP treatment with PTLs^[^ ^110^ ^]^ is still limited by the low number of existing studies. Immune cells that show high viability after direct exposure to CAP include monocytes^[^ ^107^, ^109^, ^111^ ^]^ and monocyte‐derived lineages (macrophages^[^ ^109^, ^112^, ^113^, ^114^ ^]^ and DCs^[^ ^115^ ^]^), all of which belong to innate immunity and form the mononuclear phagocyte system (MPS). Briefly, in the tissue, in the presence of different signaling molecules, monocytes can differentiate to macrophages and some DCs. While both type of cells are found in tumor bed, where they are involved in phagocytosis and production of pro‐inflammatory cytokines, DCs are primary APCs that uptake the neoantigens and migrate to the lymph nodes to prime cells of the adaptive immune system (T cells).^[^ ^116^ ^]^ Given the robustness of MPS cells, they have been the most studied immune cells in the context of CAP as a direct immunomodulator (**Table** **4**). Although macrophages and DCs are an important first line of immunologic defense, systemic and specific cancer clearance can only be ensured by adaptive immunity with T cells at its center. In addition to T cells, natural killer cells (NK cells) have been recently recognized for their innate ability to identify and kill cancer cells. Although these cells belong to innate immunity, they are of the same lineage as T cells and are functionally closer to them. Last of all, neutrophils have also emerged as important regulators of cancer. They share common progenitor with MPS cells, but their presence is usually associated with negative prognosis. Here, we summarize the current knowledge on immunomodulatory effects of CAP on these different cells of the immune system, each of which has an important role in fighting cancer.

**Table 4:** CAP could stimulate macrophage and dendritic cell functions to promote systemic anti‐tumor responses when either cancer cells (CC) or these immune cells (IC) were treated

Effect on different functions of macrophages and dendritic cells	Cells treated with CAP	CAP treatment modality
Altered surface marker expression (Differentiation, maturation, and polarization ↑, pro‐inflammatory phenotype tendency ↑)	CC	Direct:^[^ ^57^, ^117^ ^]^ (in vitro) Indirect:^[^ ^49^ ^]^ (in vitro)^[^ ^51^, ^118^, ^119^ ^]^ (in vivo)
	IC	Direct:^[^ ^109^, ^113^, ^115^, ^118^ ^]^ (in vitro) Indirect:^[^ ^110^ ^]^ (in vitro)
Altered secretory profile (Pro‐inflammatory tendency ↑)	CC	Direct:^[^ ^57^, ^117^ ^]^ (in vitro),^[^ ^55^, ^59^ ^]^ (in vivo) Indirect:^[^ ^49^ ^]^ (in vitro)
	IC	Direct:^[^ ^57^, ^109^, ^113^, ^115^, ^118^, ^120^ ^]^ (in vitro) Indirect:^[^ ^110^ ^]^ (in vitro)
Cancer killing ↑, tumorgenicity ↓	CC	Direct:^[^ ^21^, ^65^, ^66^ ^]^ (in vitro) Indirect:^[^ ^49^ ^]^ (in vitro)
	IC	Direct:^[^ ^66^, ^109^, ^112^, ^113^, ^118^ ^]^ (in vitro)
Migration ↑	CC	Direct:^[^ ^21^ ^]^ (in vitro)
	IC	Direct:^[^ ^113^, ^114^ ^]^ (in vitro)
Tumor infiltration ↑	CC	Direct:^[^ ^54^, ^55^, ^61^ ^]^ (in vivo)
		Indirect:^[^ ^48^, ^51^, ^119^ ^]^ (in vivo)
Recruitment and activation of adaptive immunity (T cells), presentation of antigens ↑	CC	Direct:^[^ ^57^ ^]^ (in vitro),^[^ ^54^, ^55^, ^61^ ^]^ (in vivo) Indirect:^[^ ^51^ ^]^ (in vivo),^[^ ^48^ ^]^ (ex vivo)
	IC	Direct:^[^ ^120^ ^]^ (in vitro)

#### 2.4.1 Macrophages

Macrophages show plasticity and, in the presence of different environmental factors, can be polarized toward a pro‐inflammatory (M1‐like) or an anti‐inflammatory, tissue‐repair (M2‐like) phenotype.^[^ ^121^, ^122^ ^]^ Macrophages found in the immunosuppressive TME are regarded as tumor associated macrophages (TAM) and are known to contribute to tumor progression.^[^ ^123^, ^124^ ^]^ Thus, TAM and the M1/M2 ratio in the context of TME represent a prognostic factor^[^ ^125^ ^]^ and a therapeutic target.^[^ ^126^, ^127^ ^]^ CAP has been reported to promote monocyte‐to‐macrophage differentiation^[^ ^109^ ^]^ and polarization toward a pro‐inflammatory, M1‐like phenotype. This was observed both for direct^[^ ^109^, ^113^, ^115^, ^118^ ^]^ and indirect^[^ ^110^ ^]^ CAP treatment of monocytes and macrophages as well as in co‐culture with direct CAP‐treated cancer cells.^[^ ^57^, ^117^ ^]^ Analysis of tumor immune infiltrates in vivo also revealed an increase in M1 (CD86, iNOS) and decrease in M2 (CD206) markers for indirect tumor treatment.^[^ ^51^, ^118^, ^119^ ^]^ For all the aforementioned experimental settings, the immuno‐secretory profile was modulated (see Table 4). While this modulation was overall pro‐inflammatory (e.g., increase in TNF*α*, IFN*γ*), the results were somewhat inconclusive and difficult to interpret. This is due to the fact that the immuno‐secretory profiles are not only very diverse and complex but also very dynamic; thus, quantitative variability can be found depending on the sampling time‐point in the study.^[^ ^117^ ^]^ While the analysis of immuno‐secretory profile is indicative of the inflammatory status and immunomodulatory effect of the treatment, functional assays (e.g., cancer killing capacity in co‐culture) should be performed to connect these results to the biological outcome. In this context, it was shown that macrophages treated with CAP^[^ ^66^, ^109^, ^112^, ^113^, ^118^ ^]^ or co‐cultured with CAP‐treated tumor cells^[^ ^21^, ^49^, ^65^, ^66^ ^]^ responded with an enhanced cancer cytotoxicity, while their cytotoxicity toward non‐cancerous cells did not seem to increase.^[^ ^112^ ^]^ It is worth remarking that suitably defining the treatment dose is of paramount importance as both pro‐ or anti‐inflammatory responses have been reported when targeting clinical applications other that cancer, that is, wound healing.^[^ ^128^ ^]^

#### 2.4.2 Dendritic Cells

Dendritic cells are phagocytotic APCs with the crucial role of priming and activating T cells to fight cancer.^[^ ^129^ ^]^ DCs are a monocyte‐derived linage and, like macrophages, have been reported to be more robust toward CAP exposure compared to lymphocytes.^[^ ^115^ ^]^ Their direct exposure to CAP was shown to favor the expression of T cell activating surface markers required for T cell activation as well as a moderate pro‐inflammatory cytokine expression.^[^ ^115^ ^]^ In co‐culture with cancer cells treated with a PTL, DCs showed enhanced maturation, cancer killing, and secretion of pro‐inflammatory signaling molecules.^[^ ^49^ ^]^ However, murine tumors treated with a PTL (in vivo) showed a tendency toward decreased DCs infiltration; although, non‐significant.^[^ ^51^ ^]^ On the other hand, direct treatment of melanoma cancer in vivo did lead to an enhanced infiltration of DCs and antigen presentation.^[^ ^54^ ^]^

#### 2.4.3 T Cells

T cells (or T lymphocytes) belong to the adaptive immune system and mediate systemic and long‐lasting tumor control and clearance. Different in vivo studies have reported increased infiltration and activation of T cells following CAP treatment of tumors^[^ ^48^, ^51^, ^54^, ^55^, ^61^ ^]^ (Table 4). This effect is likely to be mediated by an increased infiltration and activation of APCs, responsible for T cell priming, through induction of ICD in cancer by CAP, though exact delineation is difficult. For example, in a recent study that traced activation of the cancer‐immunity cycle in mice following CAP treatment of melanoma, there were more activated cytotoxic T cells (ICOS/PD1/IFN*γ* markers) both in the TME as well as in the tumor‐draining lymph node.^[^ ^54^ ^]^ However, it could not be differentiated whether this was in response to CAP treatment alone or CAP‐induced ICD of the melanoma cells. Namely, in addition to ICD, CAP can also act via other mechanisms to enhance T cells activation. For example, it could enhance the ability of the treated macrophages to activate antigen‐specific T cells.^[^ ^120^ ^]^ So far, there are not many studies that investigated the direct functional effects of CAP on T cells by exposing these immune cells to a CAP source or a PTL. The reports on this issue mainly conclude on the sensitivity of lymphocytes to CAP exposure.^[^ ^107^, ^108^, ^115^ ^]^ However, there is first evidence that short exposure of T cells to CAP can not only preserve their viability but also augment the release of pro‐inflammatory IL‐2 and IFN‐*γ* molecules in the presence of costimulatory signals. This was observed for both T cells that were isolated from murine lymph and then treated with CAP as well as for T cells that were left in their microenvironment (lymph node) and treated there.^[^ ^120^ ^]^ In addition, the authors reported adoptive T cell transfer, where cytotoxic (CD8+) T cells isolated from ex vivo CAP‐treated lymph node were injected into tumor‐bearing mice, which resulted in a strong anti‐tumor effect.

#### 2.4.4 Natural Killer Cells

Natural killer (NK) cells belong to the family of innate lymphoid cells. From a functional perspective, their adaptive immunity analogues are cytotoxic (CD8+) T cells as NK cells can specifically recognize infected and malignant cells and provide direct cytotoxicity.^[^ ^130^ ^]^ However, in contrast to T cells, they do not require priming by APCs, which enables a much quicker antitumor response. It is to say, they have innate ability to kill cancer. That is, NK cells express a number of activating and inhibiting receptors on their surface, which can interact with target ligands on the cells, and this balance between activating and inhibitory interactions determines if a cytotoxic response is initiated. In addition, NK cells secrete different cytokines such as IFN*γ* and TNF*α* to promote innate and adaptive immune responses.

Due to their innate ability to eliminate cancerous cells, NK cells have been gaining increased interest and are being widely explored in the context of cancer immunotherapies.^[^ ^130^, ^131^ ^]^ However, their clinical success in solid cancers is challenged by strong immunosuppression, and to overcome this, different strategies such as ex vivo pre‐conditioning and engineered NK cells, have been employed. The effects of CAP on NK cells and their responses to the CAP‐treated cancer cells have only begun to be explored and more studies are required. Clemen et al. published the first reports showing that CAP treatment is able to increase NK cell activating ligands on the cancer cell surface.^[^ ^132^ ^]^ In this study, it was reported that in co‐culture with such CAP‐treated skin cancer cells, NK cells respond with higher cytotoxicity and altered immuno‐secretory profile, for example, enhanced IL‐6 and IL‐8, which could act as pro‐inflammatory and chemotactic molecules, respectively. Importantly, CAP treatment of non‐malignant keratinocyte skin cells did not increase surface NK cell activating ligands and did not enhance the cytotoxicity of NK cells toward these cells. Taken together, these represent promising initial results that should receive more attention.

#### 2.4.5 Neutrophils

Neutrophils belong to innate immunity and can neutralize their target through several mechanisms: phagocytosis, release of intracellular granules, and formation of neutrophil extracellular trap (NET). Neutrophils were observed to be sensitive to CAP exposure and to respond with the formation NET followed by enhanced release of neutrophil‐attractant IL‐8.^[^ ^108^, ^133^ ^]^ While NET is of relevance in areas such as wound healing due to its anti‐microbial function, it is shown to have pro‐tumorigenic effect.^[^ ^134^, ^135^ ^]^ It is generally acknowledged that neutrophiles can promote cancer invasiveness and progression.^[^ ^136^ ^]^ Like macrophages, neutrophils also show plasticity and can be reprogrammed by the TME. As different CAP treatment regimens could lead to differential responses in neutrophils,^[^ ^137^ ^]^ it would be interesting to analyze this in the context of cancer treatment.

## 3 CAP Treatment Modalities in Clinics

### 3.1 Limitations of Direct CAP Treatment and Plasma‐Treated Liquids

Although CAP represents a promising cancer therapy that could promote durable anti‐tumor immunity and act as an adjuvant in combination with existing treatments, current CAP treatment modalities (direct treatment and indirect via PTLs) have several critical limitations in terms of clinical application for different cancers.^[^ ^138^ ^]^ Direct CAP treatment is currently limited to the treatment of tumors and lesions that are easily accessible with the device. This is why clinical studies with CAP published up to now have only included patients with locally advanced head and neck squamous cell carcinomas or with pre‐malignant skin lesions that can develop into squamous cell carcinoma (actinic keratosis).^[^ ^139^, ^140^ ^]^ One approach to increase the penetration depth of direct CAP treatment was reported in a recent in vivo study. In this study, Chen et al., demonstrated the use of a hollow transdermal microneedle patch to improve the delivery of CAP treatment deeper into melanoma tumors using a subcutaneous mouse model.^[^ ^34^ ^]^ Such strategy decreased tumor volume and increased mouse survival compared to direct CAP exposure onto the tumor and CAP treatment using a solid microneedle patch (an additional control group). Furthermore, they combined this treatment with an immune checkpoint inhibitor (*α*PD‐L1), which led to more pronounced tumoricidal and survival benefits, accompanied by a higher immune response and potential abscopal effects. While this important study provided a solution to improve the penetration depth of direct CAP treatment into the tumor, it still relied onto direct access to it with the device. At the same time, it has been suggested that direct CAP treatment could be useful in combination with surgery to treat the resection margins of deeper tumors, but these studies have yet to be performed and validated.

To reach tumors inside the body, additional engineering strategies have been suggested to widen the range of cancer applications with CAP. Several groups have studied the possibility of developing endoscopic CAP devices for direct treatment within body cavities.^[^ ^141^ ^]^ Robert et al. developed an endoscopic CAP source which allowed for CAP to propagate along a dielectric capillary and reach tumors in several orthotopic tumor mouse models.^[^ ^142^ ^]^ Helium and neon feed gas were used to directly treat colon tumors and pancreatic tumors, respectively, and in both cases, the authors observed a decrease in tumor volume based on bioluminescence imaging analysis. This was achieved without significant side effects such as burning or bleeding at the CAP treatment site. In addition, the authors attempted the use of their endoscopic CAP source to treat tumors in the mouse lung. Though anti‐cancer effects were not observed in this tumor model, the authors report that the use of helium was very disturbing for the mice under anesthesia, while neon was tolerable. Taken together, it is clear that the endoscopic CAP source provides an exciting engineering solution to deliver direct CAP treatment to tumors within the body, but further optimization and considerations per cancer type are still needed.

In contrast to direct CAP treatment, PTLs are minimally invasive as they can be injected and repeatedly administered to deeper tumor tissues. In fact, several studies in Japan have demonstrated the ability of PTLs to reduce metastatic ovarian tumor nodes in mice following PTL perfusion. In an important study, Utsumi et al. demonstrated that local injection of PTLs was able to inhibit tumor growth of subcutaneous chemo‐resistant ovarian cancer cells in mice.^[^ ^143^ ^]^ Later, the group followed up the study and reported that perfusion of PTLs into the mouse abdominal cavity could suppress intraperitoneal metastasis of ovarian cancer^[^ ^144^ ^]^ and even stimulate proinflammatory macrophage infiltration.^[^ ^119^ ^]^ Nevertheless, by using PTLs to treat the target, several potentially therapeutic components of CAP are lost, and never reach tumor. That is, in direct treatment, the treated tissue is exposed to all the physical (e.g., high electric fields, ultraviolet radiation) and chemical components (e.g., free radicals, neutral molecules) of CAP. For indirect treatment; however, all the physical aspects of CAP are removed from treatment and only a limited number of RONS remain, namely the long‐lived RONS (e.g., H_2_O_2_, NO_2_^−^, NO_3_^−^, and ONOO^−^). There are several studies that highlighted the importance of CAP‐generated short‐lived RONS during direct treatment (e.g., •OH, O, and •NO).^[^ ^56^, ^145^ ^]^ Due to the complex environment generated by CAP, it is difficult to delineate potential synergistic effects between different physical and chemical CAP components.^[^ ^65^, ^146^ ^]^ Therefore, it remains an open question whether the mechanisms of action of direct and indirect treatment are the same in terms of anti‐cancer effects; though, both modalities have demonstrated immunotherapeutic properties.

Not only are potentially crucially short‐lived RONS lost in PTL cancer treatment but PTLs can also be easily diluted and washed away by fluids within the body. Indeed, a study investigating the lifetimes of several long‐lived RONS in blood plasma and in processed whole blood, have demonstrated that different species are quenched by different components of blood.^[^ ^78^ ^]^ While H_2_O_2_ was stable in PBS, it was significantly scavenged by blood plasma immediately, potentially due to presence of dissolved proteins and lipids (e.g., albumin, globulins, fatty acids, and cholesterol), and completely scavenged within 30 s in whole blood. In contrast, reactive nitrogen species (e.g., NO_2_^−^, NO_3_^−^, •NO) were stable in blood plasma up to 5 min but continually decreased in whole blood. On the other hand, ONOO^−^ was scavenged within 60 s after CAP treatment in both blood plasma and whole blood. Taken together, it is clear that intravenous delivery of PTLs for therapy is not a likely option as reactive species are lost to the blood in transit. In this context, the location of the PTL injection site in relation to the tumor target is critical for therapeutic effect and must be optimized.

Altogether, current CAP treatment modalities can find a niche within oncotherapy, but different engineering solutions and deeper fundamental insights are needed to improve their limitations and broaden their application. Whether it is with direct treatment with DBDs, APPJs, or newly engineered CAP devices such as the endoscopic CAP, one of the biggest needs in the field is to quantify the degree or amount of CAP treatment: the “plasma treatment dose.” Depending on the pathology to be treated, different plasma treatment doses will be required, and defining the right treatment dose or therapeutical window will allow the treatment to be standardized and predictably elicit the desired anti‐cancer effect. Furthermore, it is evident that there is also a need for new and innovative modalities, that can broaden and improve clinical application of CAP. Here, we propose the use of PTHs as a way of hybridizing the advantages of direct CAP and PTLs for localized and minimally‐invasive treatment of deeper tumor tissues. Moreover, PTHs can also function as a convenient platform for more advanced treatment strategies, as will be discussed in the next sections.

### 3.2 Perspective on Plasma‐Treated Hydrogels ‐ A Novel CAP Modality

**Figure 3:**
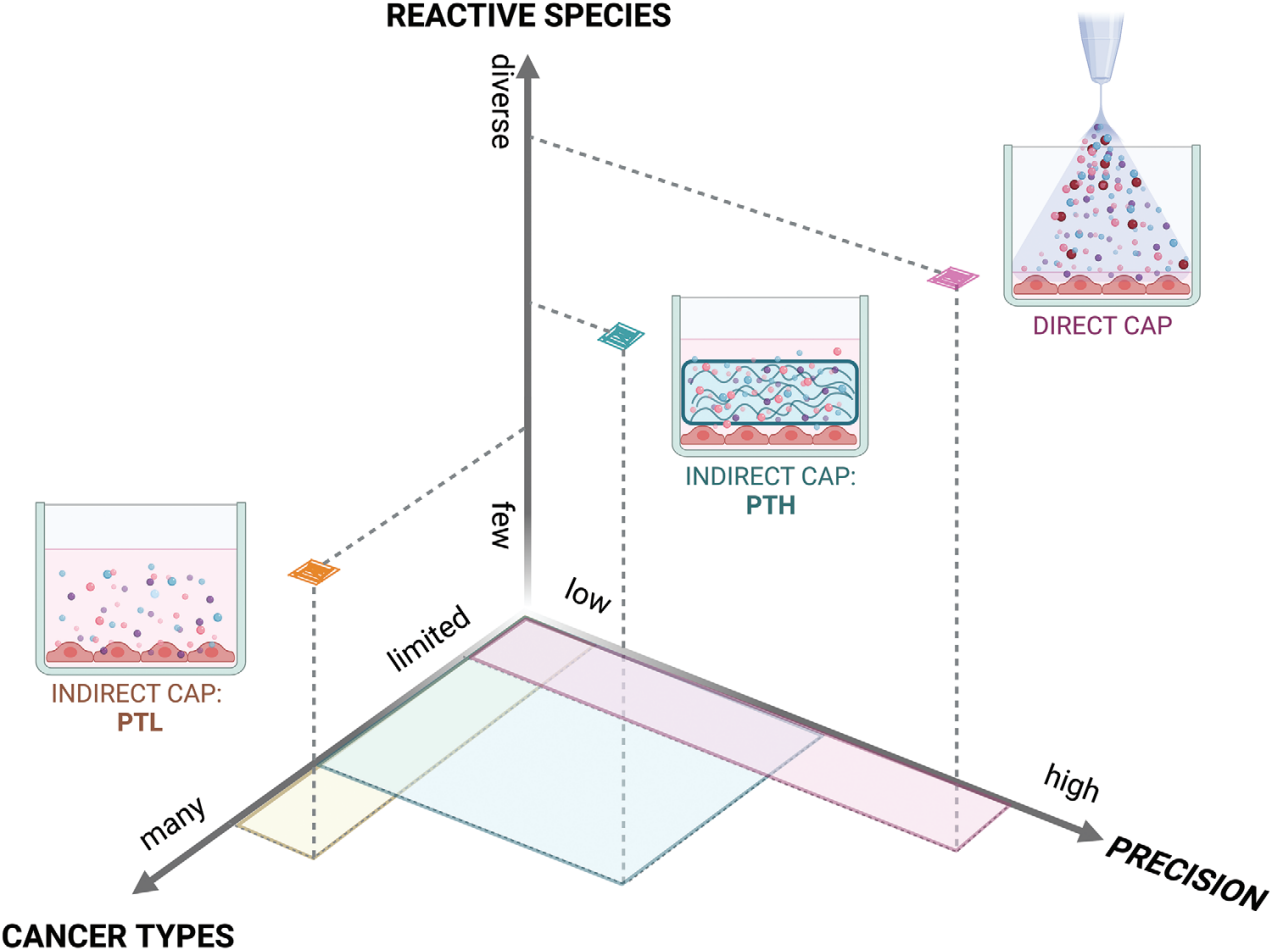
Plasma‐treated hydrogels (PTHs) are a novel CAP treatment modality. PTHs offer a way of combining the advantages of current treatment modalities: high local RONS‐delivery of direct CAP treatment and minimally‐invasive RONS‐delivery of plasma‐treated liquids (PTLs). PTHs can also be injectable, yet, more viscous and chemically complex than PTLs. Figure created with Biorender.

Hydrogels are water‐swollen, 3D, porous networks made up of crosslinked hydrophilic polymers.^[^ ^147^ ^]^ They show excellent biocompatibility and have been explored for decades for their clinical utility, ranging from ocular applications and wound and tissue regeneration to the delivery of cancer drugs.^[^ ^148^ ^]^ Their versatility, ease of manipulation and tailoring, and ability to provide protection to cargo make hydrogels an attractive platform for spatiotemporally controlled therapeutic delivery of small molecules, macromolecules, and cells. To this end, our group began investigating plasma‐treated hydrogels (PTHs) as vehicles for therapeutic RONS delivery in cancer.^[^ ^35^, ^149^, ^150^ ^]^ Keeping in mind the aforementioned clinical limitations of current CAP treatment modalities, we postulated that using hydrogels instead of liquids to indirectly treat tumors within the body can be an advantage. Namely, with PTHs, we could potentially achieve higher local concentrations and longer local delivery of therapeutic RONS molecules, while maintaining low invasiveness of the treatment procedure, as hydrogels can also be engineered to be injectable (**Figure** **3**). Hydrogels are more viscous than liquids, which allows for better material retention upon injection, and can crosslink after minimally invasive injection to the target site. In contrast to liquids, the varied chemistry of polymers provides potential for modified reactivity with plasmas and they are likely to form organic peroxides; so, PTHs might also allow for fine‐tuning and/or enhanced and prolonged RONS generation and release.

In addition, as hydrogels are traditionally used for controlled drug delivery and tissue engineering, PTHs represent an intuitive platform to combine CAP‐based therapies and hydrogel‐based drug delivery or regenerative medicine. This may not only improve the CAP treatment efficacy but also help its translation to the clinic.

Through the optimization of hydrogel design (e.g., polymer(s) type and concentration, chain length and rigidity, crosslinking method etc.) and engineering strategies (chemical functionalization of polymer), the physicochemical properties of hydrogels can be easily adjusted to achieve the desired mechanical properties, drug release profile, and bioactivity.^[^ ^151^, ^152^ ^]^ For instance, simply by increasing the concentration of the polymer or crosslinking agent, denser hydrogels with higher mechanical properties, lower swelling degree (volume change), and smaller pore sizes can be obtained to delay the release of cargo molecules.^[^ ^153^ ^]^ That is, the pore or mesh size determines the drug/molecule release kinetics. Very small molecules (e.g., RONS) will be quickly released to the surrounding media. The more similar the molecule size is to the mesh size, the slower it is released. If the drug molecule is larger than hydrogels pores, then it cannot be released through diffusion anymore, but is gradually liberated with hydrogel degradation, mechanical deformation, or swelling.^[^ ^152^ ^]^ Beside physical hydrogel properties, chemical interactions of hydrogel with the drug can be used to slower the drug release from the hydrogel. These interactions can be strong covalent interactions; so that the drug is released only upon hydrogel degradation or bond cleavage, or they can be charge‐based or hydrophobic interactions that provide relatively weaker binding domains to prolong retention of the drug independent of the mesh size.^[^ ^152^ ^]^ Apart from sustained drug delivery, hydrogels can also function as tissue‐specific scaffolds in tissue engineering, where they are used to mimic the biological composition and physicochemical properties of extracellular matrix of the respective body tissue. In this context, hydrogels can provide chemical and biomechanical signals to the surrounding cells to promote their proliferation and/or guide differentiation mechanisms, which are the key processes in the tissue regeneration and repair. For example, hydrogels that incorporate collagen and hydroxyapatite to mimic the organic and inorganic phase of the bone, respectively, are used in bone healing where they promote differentiation of mesenchymal stem cells to osteoblasts; and thus, bone growth.^[^ ^154^ ^]^

**Figure 4:**
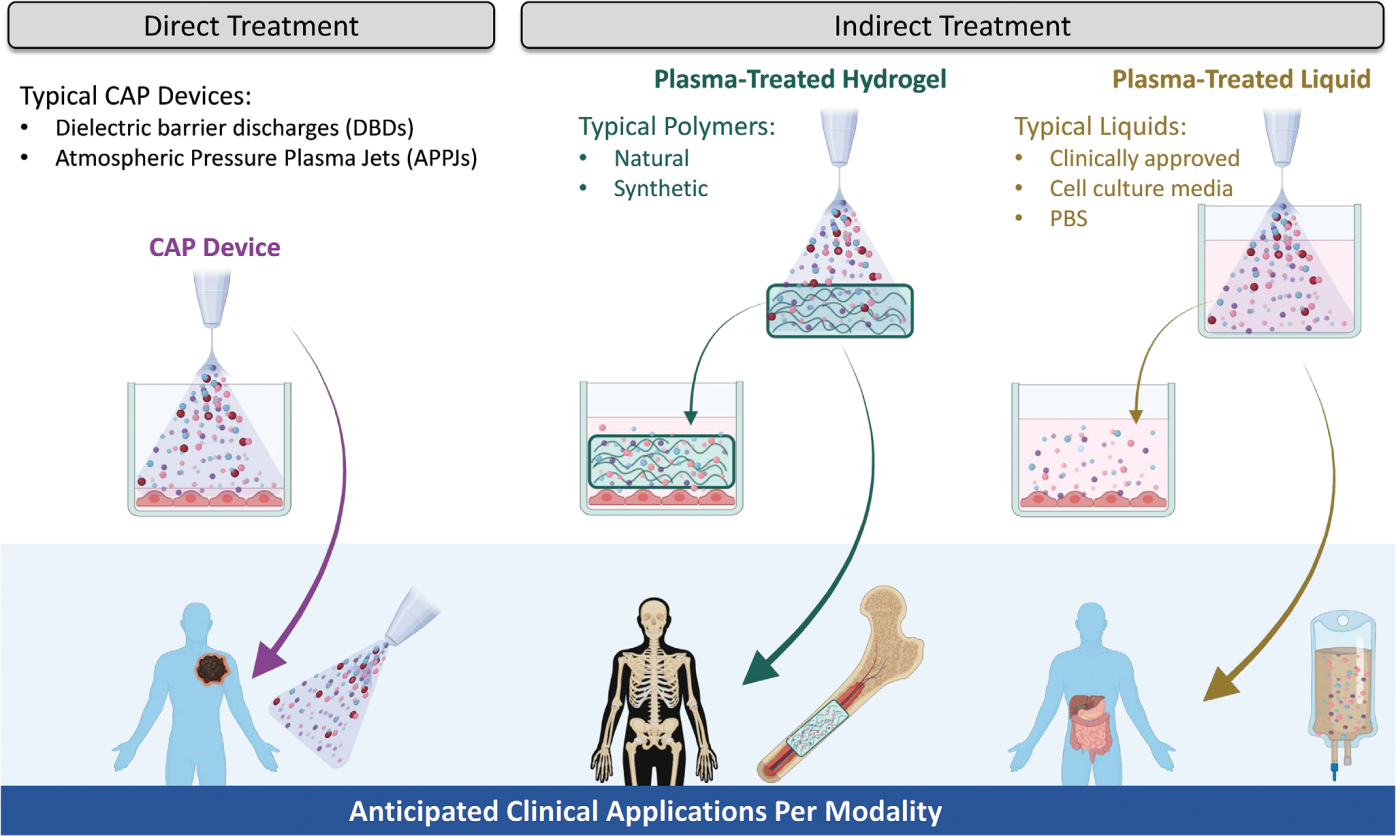
PTHs can broaden the clinical utility of CAP treatment as they offer a minimally‐invasive method for precise and prolonged treatment of non‐surface tumor tissues. Beside delivery of RONS, PTHs could incorporate a second functionality as tissue‐specific scaffolds or carriers of a further (immunomodulatory) therapeutic agent for more efficient and advanced combinatorial treatment strategies. Figure created with Biorender.

Therefore, PTHs could serve not only as vehicles for RONS delivery to treat tumors but could also incorporate further drugs^[^ ^155^, ^156^ ^]^ to enhance anti‐tumor and immune‐stimulatory effect of RONS or be used to promote tissue‐repair for post‐operative cancer treatment or wound healing applications. Combinatorial treatment approaches are particularly relevant in cancer treatment because disease progression often involves multiple, interconnected pathways, and is characterized by treatment resistance or immune evasion. For instance, it has been shown that the combination of chemotherapy drugs with PTL can allow reducing drug dose, with the potential associated benefits for the patient.^[^ ^158^ ^]^ As CAP could assist the cancer‐immunity cycle and also sensitize cells to the treatment,^[^ ^157^ ^]^ combination with immunotherapies already in use such as immune checkpoint inhibitors,^[^ ^34^ ^]^ or even with low doses of chemotherapeutics can be of interest to improve the therapeutic outcomes. Taken together, PTHs could significantly broaden the possible clinical application and utility of CAP for the treatment of different tumors (**Figure** **4**).

### 3.3 Plasma‐Treated Hydrogels: Proof of Concept

The concept of PTHs is relatively new; so, this section reviews the main findings reported up to now, that could pave the way for future developments. PTHs are not to be confused with the hydrogels that have been used as surrogate tissue models to study the penetration depth and interaction of CAP with biological tissues.^[^ ^159^ ^]^ Such hydrogels are crosslinked; and thus, in a hydrated solid before their exposure to CAP. They are meant to mimic the physicochemical properties and behavior of biological tissues in contact with direct CAP treatment.^[^ ^160^ ^]^ In contrast, PTHs are conceived as delivery platforms for CAP‐generated RONS. In this case, the hydrogels are exposed to CAP in their non‐crosslinked state, as aqueous polymeric solutions. After CAP treatment, the polymeric solution is crosslinked to form a PTH, hereby evenly encapsulating the RONS within the 3D hydrogel network. Once in contact with the target tumor, small RONS molecules can diffuse from the aqueous phase of PTHs to the surrounding tissues.

For PTHs, the chemistry of the different polymers used in hydrogel design is critical as it determines the interactions of the reactive species from the plasma phase with the liquid phase. This interaction between polymer chains in solution and CAP may give rise to different outcomes: scavenging of RONS or generation of secondary RONS through the reaction of the chemical groups in the polymer with RONS from CAP or within the liquid. All this can modify the kind and concentration of RONS generated in the polymeric solution (in contrast to, for instance, water or a saline solution). Beside, this reactivity could also alter the polymer's physicochemical properties in different ways (e.g., fragment it, or generate chemical by‐products, modify its mechanical properties and crosslinking ability, etc.). For these reasons, interaction of each individual polymer with CAP should be carefully assessed. To this end, different polymers have been considered to date, including alginate,^[^ ^35^ ^]^ gelatin^[^ ^149^ ^]^ and their combination, methylcellulose,^[^ ^150^ ^]^ polyethyleneoxyde‐based copolymers,^[^ ^161^ ^]^ and polyethyleneglycol–polylactide copolymers.^[^ ^162^ ^]^ In all cases, RONS were successfully generated within the polymer solutions in a CAP treatment time dependent manner, and the polymers maintained the ability to crosslink and form self‐standing hydrogels (through different crosslinking methods including ionic crosslinking, thermal gelling, or UV crosslinking).

Interestingly, some of the biopolymers (e.g., alginate and gelatin) showed the ability of buffering the solution and maintaining the pH stable even following long CAP treatment times. This contrasts with CAP treatment of conventional unbuffered liquids (e.g., water, saline solutions), where acidification occurs, which promotes reactions in which biologically active RONS are depleted and peroxinitrous acid is formed.^[^ ^3^ ^]^ In this regard, it is important to highlight that the stability of the RONS formed is highly dependent on the chemistry of the hydrogel. For example, H_2_O_2_ was stable in CAP‐treated gelatin solution for up to 72 h, while NO_2_^−^ showed a decrease between 20% and 40%.^[^ ^149^ ^]^ In contrast, in CAP‐treated methylcellulose solutions, H_2_O_2_ and NO_3_^−^ showed a certain decrease while NO_2_^−^ remained essentially unaltered.^[^ ^150^ ^]^ Beside, the crosslinking process could also influence the concentration of RONS initially generated by the CAP treatment. It was reported that for methylcellulose PTH that was gelled thermally, NO_2_^−^ and NO_3_^−^ remained essentially unchanged, but the concentration of H_2_O_2_ strongly decreased.^[^ ^150^ ^]^ Thus, careful case‐to‐case analysis is essential to relate the RONS generated and maintained after the PTH crosslinking to their biological activity.

Up to now, at the polymer concentrations required to form stable hydrogels, no major modifications have been observed in the alginate or gelatin hydrogels as recorded by SEM microscopy and FTIR‐ATR spectroscopy.^[^ ^35^ ^]^ However, it must be noted that at lower polymer concentrations, which would not be suitable for the formation of the respective hydrogels, CAP was observed to cleave bonds in the macromolecules, resulting in lower molecular weight polymeric units.^[^ ^150^, ^161^ ^]^

The biological effects of PTHs reported up to now consistently show the same efficacy as PTLs, with PTHs being able to selectively kill osteosarcoma cell lines.^[^ ^35^, ^150^ ^]^ Furthermore, recent in vivo studies provided first evidence for both the clinical safety of PTHs using gelatin/alginate PTH in combination with hydroxyapatite scaffolds to support bone formation^[^ ^163^ ^]^ and for clinical efficacy of PTHs for the treatment of post‐surgical, residual cancer cells.^[^ ^162^ ^]^ In the latter, Zhang et al. treated a synthetic polymer ([Poly‐DL‐lactide]–[poly‐ethylene glycol]–[poly‐DL‐lactide]) for 20 min with CAP to obtain an injectable, thermosensitive PTH. Upon surgical removal of bladder cancer, the PTH was applied, and the wound was closed. Such combinatorial treatment approach was reported to be very effective as all mice from this treatment group survived to the end of the study. While the authors demonstrate that the anticancer effects of PTH act through increased intracellular RONS and decreased NADH using 3D spheroid models, the immunogenicity of PTH treatment remains to be studied. As PTHs are still a novel concept, further research is needed to demonstrate biological effects and efficacy of different PTHs for cancer treatment and anti‐tumor immunity.

### 3.4 Plasma‐Treated Hydrogels: Future Challenges and Directions

**Figure 5:**
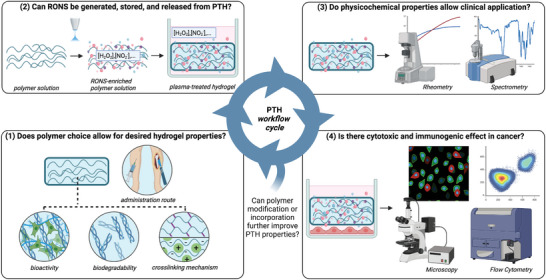
Proposed research workflow cycle for developing plasma treated hydrogels (PTHs). The key properties of PTHs to be tested are: efficacy of RONS delivery, clinical safety and utility (e.g., no toxic chemical by‐products and sufficient mechanical strength), and functionality in treating cancer. Figure created with Biorender.

There are many considerations behind the design of PTHs, which require expertise both in biomaterials and plasma medicine, including the possible regulatory classification of this novel product. In **Figure** **5**, we propose the following research workflow cycle for the development of PTHs:

1. Choosing a polymer(s) that allows for desired hydrogel properties, for example, injectability and biodegradability; choosing a concentration to reach a compromise between viscosity not being too high (to enable uniform reaction with CAP) and a hydrogel that has good mechanical properties;
2. Characterization of RONS generation in the chosen polymer system, their stability and release from the PTH; in relation to this, ensuring that the methods and protocols that are commonly applied to quantify CAP‐generated RONS in aqueous solutions (PTLs) are also suitable for the selected polymeric solutions;
3. Physicochemical characterization of the PTH, especially with regard to potential CAP‐induced chemical modifications, as this could influence the clinical safety and mechanical properties of the PTH. Note that desired mechanical properties of a hydrogel might be different for different tissues and shear stress exposures;
4. Functional characterization of PTH, for example, cytotoxicity to cancer cells and non‐malignant cells, different tumorigenic and immunogenic biological effects

Of course, the last step should involve iteration of the “PTH workflow cycle” to possibly tune treatment conditions, polymer concentration, add further polymers or biomolecules to the system, or introduce chemical modification to the chosen ones in order to improve RONS generation, bioactivity, or therapeutic effect of the PTH.

In this sense, we may point out three further future focuses in PTHs research. First, different polymers should be explored in the context of RONS generation and storage (including organic peroxides and secondary RONS). This means, different polymers might allow for superior generation and release of RONS. For example, we showed that in comparison to alginate, gelatin was able to generate significantly more RONS across comparable conditions and CAP sources, implying that protein‐based polymers might be particularly effective for PTHs.^[^ ^149^ ^]^ Second, PTHs up to now have been generated by employing APPJs, where the CAP gas interfaces with the polymer solution surface on a very small area. In this context, it would be worth exploring alternative plasma sources such as dielectric barrier discharge (DBD) devices, that can allow treatment of larger surface areas of the polymer solution which might maximize the production of RONS. However, different plasma sources (and carrier gases) could also introduce different physical and chemical modifications to the polymers. Thus, accessing both of these aspects (concentration of RONS and physicochemical properties of PTH) will help find the most adequate plasma sources for generation of functional PTHs. Third, it should be investigated if PTHs could/should incorporate a second function beside delivery of RONS, without compromising their ability to act as vehicles for RONS and kill cancer cells, but also, without this second function being compromised by the RONS. It is to say, whether PTHs can also be used to deliver a further immunomodulatory agent for an enhanced therapeutic effect or if PTHs can also provide biological and mechanical scaffold that would promote survival and proliferation of non‐malignant cells.

As a guide for the development of different PTHs, **Table** **5** summarizes several key starting considerations when designing PTH and choosing the polymer that will be used to obtain the hydrogel, according to the workflow in Figure 5. First, regarding the choice of a polymer that will be used to obtain the hydrogel. To begin with, it is important to take into account the crosslinking mechanism of the polymer, both in the context of the invasiveness of PTH delivery and compatibility of CAP with it. Depending on the crosslinking mechanism, hydrogels can be divided into chemical or permanent hydrogels and physical or reversible hydrogels.^[^ ^164^ ^]^ Chemical hydrogels are formed through covalent bonds (e.g., photopolymerization or enzymatic crosslinking); and thus, have higher mechanical properties and stability compared to physical hydrogels. Their gelation and different physicochemical properties are also more easily controlled and fine‐tuned. However, chemically cross‐linked hydrogels require the presence of reactive chemical groups and CAP‐generated RONS or UV light might easily interact with them, either consuming the crosslinking agents or initiating the crosslinking process already during CAP treatment. On the other hand, physical hydrogels are formed through a large number of non‐covalent interactions (e.g., ionic or supramolecular interactions) and can easily be obtained using biomolecules. Among different hydrogels, the ones that can be injected represent the most versatile therapeutic platform as they allow for minimally‐invasive administration and can adapt their shape to different cavities and geometries in the body.^[^ ^165^, ^166^, ^167^ ^]^ Injectable hydrogels with in situ polymerization and quick sol–gel transition are very attractive as they allow for stable control over gelation kinetics and improve material retention, which minimizes the loss of hydrogel's mechanical properties and/or loaded molecules.^[^ ^168^ ^]^ An injectable hydrogel should be viscous enough to be pushed through a catheter/needle system with a syringe, but should also exhibit good elastic or solid‐like behavior to ensure that the injected volume remains at the target site and can locally deliver therapeutic agents. For example, there is a couple of clinical trials (NCT02891460, NCT02307487) that used thermosensitive biodegradable hydrogel (composed of Pluronic F‐127, Polyethylene glycol‐400, and hydroxypropyl methyl cellulose) to deliver antitumor drug mitomycin to patients with bladder cancer. Thermosensitive hydrogels have high viscosity (gel‐like state) at body temperature and low viscosity (liquid state) at lower room temperatures, which allows drug incorporation and injection at liquid state and sustained drug release in the body. Further commonly employed strategies to achieve injectability of a hydrogel are use of a device that mixes the polymer with crosslinker agent at the very moment of injection, triggering the formation of the hydrogel in the body, or by use of polymer solution with shear‐thinning behavior, where the stress from pushing the needle transiently lowers their viscosity to allow injection. Besides the cross‐linking mechanism, other important properties that should be considered when choosing the optimal polymer for PTH include biodegradability, bioactivity, and chemical reactivity (Table 5). Biodegradability is one of the most interesting features and should be tuned according to the required therapeutic dosage in order to allow a timely degradation of the hydrogel and repeated administration. Last of all, all these properties can be further influenced through concentration and molecular weight of polymer(s) and number of functional groups or crosslinking degree as these affect swelling, porosity, and mechanical properties of the hydrogel.

**Table 5:** The choice of polymer could influence different functional properties of PTH

Polymer property	Influenced PTH property → Functional consequences	
Crosslinking mechanism	Administration route of the hydrogel (injection or implantation)	Invasiveness of the procedure, complexity of administration, need for specific devices that would trigger the crosslinking reaction
	Gelation time	Stability of the hydrogel within the target site (and long‐lived RONS therein)
	Mechanical and viscoelastic properties (*These can be further fine‐tuned by modifying concentration of the polymer or crosslinking degree*)	Physical stimuli for the cells, stability, resistance to the shear stress
Similarity to the extracellular matrix	Bioactivity (*Note that natural polymers (biopolymers) with highest bioactivity do not offer high mechanical properties, and their physicochemical properties are not easily tunable*. *Bioactivity can be further finetuned by functionalization of polymer, for example, with cell‐adhesion peptide sequences*.)	Cell adhesion, proliferation, differentiation; tissue regeneration (*Note that having high bioactivity might reduce the cytotoxic effect of PTHs as it could help cancer cells survive the treatment. However, PTHs with bioactive polymers could be useful for post‐operative cancer treatment or in wound healing*.)
Biodegradability	Stability and removal route	Invasiveness of the removal procedure, treatment duration time and possible frequency of hydrogel administration, drug release kinetics
Reactive chemical groups	Interaction with CAP and RONS (*This can be further fine‐tuned by modifying concentration of the polymer*. *Note that protein‐based polymers are seen to generate more RONS on example of gelatin, but are generally expected to have higher (oxidative) interaction with CAP*.)	Polymer fragmentation (mechanical properties), type and amount of generated RONS and RONS scavenging, pre‐crosslinking, generation (and toxicity) of chemical by‐products
	Possibilities for functionalization	Bioactivity, interaction with loaded drug and release kinetics
Biocompatibility (after exposure to CAP)	Immunogenicity and toxicity	Pro‐inflammatory immune responses

To sum up, PTHs offer a powerful platform to lead CAP‐based cancer therapy to the next level. As discussed in this work, the current state of the art opens the door for further translational work of CAP technology in onco‐immunotherapy. It is now on experts in biomaterials and plasma physics, chemists, biomedical engineers, oncologists, and immunologists working in this field to get together and bridge this gap to the clinics.

## Conflict of Interest

The authors declare no conflict of interest.
